# Vitamin D Status Is Negatively Related to Insulin Resistance and Bone Turnover in Chinese Non-Osteoporosis Patients With Type 2 Diabetes: A Retrospective Cross-Section Research

**DOI:** 10.3389/fpubh.2021.727132

**Published:** 2022-02-11

**Authors:** Jie Zhang, Yangjun Li, Dong Lai, Di Lu, Zhenhao Lan, Junfei Kang, Yidong Xu, Shaofang Cai

**Affiliations:** ^1^Xiamen Second Hospital Affiliated Xiamen Medical College, Xiamen, China; ^2^Hwa Mei Hospital, University of Chinese Academy of Sciences, Ningbo, China

**Keywords:** vitamin D, insulin resistance, bone turnover, type 2 diabetes, Chinese non-osteoporosis patients

## Abstract

**Background and Objectives:**

Vitamin D status is closely related to blood glucose and bone metabolism in patients with type 2 diabetes (T2DM). Vitamin D affects bone density and bone metabolism, leading to osteopenia and osteoporosis. Insulin resistance increases the risk of osteoporosis in patients with T2DM. Our previous studies have shown a negative correlation between insulin resistance and 25-hydroxy vitamin D [25(OH)D] levels. The aim of the present study was to determine the association between vitamin D status and insulin resistance and bone metabolism in patients with T2DM.

**Subjects and Methods:**

A retrospective cross-section research was carried out among 109 non-osteoporosis patients with T2DM. Their fasting blood glucose (FBG), 25(OH)D, fasting blood insulin (FINS), glycosylated hemoglobin (HbA1c), serum creatinine (SCr), calcium (Ca), phosphorus (P), insulin-like growth factor-1 (IGF-1), bone alkaline phosphatase (BALP), body mass index (BMI), glomerular filtration rate (eGFR), homeostatic model estimates of insulin resistance (HOMA-IR), and calcium-phosphorus product were measured routinely.

**Results:**

Both in men and women, 25(OH)D was negatively correlated with BALP (β = −0. 369, *p* ≤ 0.001)and HOMA-IR (β = −0.349, *p* ≤ 0.001), and positively associated with IGF-1(β = 0.672, *p* ≤ 0.05). There was a negative correlation between HOMA-IR and IGF-1 (β = −0.464, *p* ≤ 0.001), and a positive correlation between HOMA-IR and BALP (β = 0.344, *p* ≤ 0.05), adjusted by confounding factors.

**Conclusion:**

Our study demonstrates that 25(OH)D concentrations are negatively correlated with insulin resistance and bone turnover. Insulin resistance increases with the decrease of 25(OH)D concentration, which can enhance bone turnover, and increases the risk of osteoporosis in non-osteoporosis patients with T2DM. This is the first study to clarify the relationship between serum vitamin D status, insulin resistance, and bone metabolism in non-osteoporosis patients with T2DM in China.

## Introduction

Both osteoporosis and diabetes are metabolism diseases, which increase rapidly in the world, especially in Asia. In Asian countries, the predicted prevalence of diabetes by the year 2030 is more than double-rates that in 2000 (1). Hip fractures occurring in Asia will be responsible for 50% of that in the world by the year 2050 in forecast reports (2, 3). Diabetic osteopathy is a complication that leads to the decrease of bone formation and bone density and increases the risk of fracture healing difficulty. Patients with diabetic osteopathy show increased osteoclastogenesis and decreased osteoblastogenesis. Accumulated research studies have shown that vitamin D deficiency was commonly found in patients with type 2 diabetes (T2DM) (4–6). It is generally agreed that vitamin D is responsible for maintaining normal levels of serum Ca and P. Therefore, subjects with vitamin D deficiency are at high risk for osteoporosis. Diabetic osteopathy has a high incidence and delay healing, which is a result of disabilities and morbidity (7).

The prediction and early intervention of osteoporosis are essential to patients with diabetes-related disorders. Insulin resistance is the basic pathologic change in patients with T2DM, which raises the risk of osteoporosis. Based on the negative correlation between 25-hydroxy vitamin D [25(OH)D] concentration with insulin resistance found in our research before (8), we boldly hypothesized that vitamin D status may be negatively related to both insulin resistance and bone metabolism in patients with T2DM. There is limited information about the relationship between vitamin D levels, insulin resistance, and bone metabolism in patients with T2DM. This study determined the relationship between serum vitamin D levels, bone metabolism, and insulin resistance in Chinese non-osteoporosis patients with T2DM. Informed consents were unnecessary, due to the retrospective and the anonymously data-analyzing.

## Subjects and Methods

### Subjects

Based on Levey et al.'s research (9), we identified 109 patients with T2DM without osteoporosis (10), who were treated in the outpatient Department of Endocrinology and Metabolism of Xiamen Second Hospital from January 1, 2014 to March 31, 2014, considering the effect of different seasons to vitamin D status. We followed the methods of Zhang et al. (8). Inclusion criteria were as follows: (i) age ranging from 20 to 70 years, (ii) history <10 years, (iii) serum parathyroid hormone concentration ranging from 15.0 to 65.0 pg/ml, (iv) a serum calcium (Ca) concentration <2.45 mmol/L, (v) normal routine blood tests of liver function, serum creatinine, serum urea nitrogen (BUN), and normal electrolytes, and not being treated with insulin or with a thiazolidinedione (TZD), estrogen, glucocorticoids, vitamin D, or drugs modulating vitamin D efficacy. The definition of osteoporosis was based on assessments of bone mineral density (BMD).

Major reasons for excluding individuals included (i) absence of T2DM or presence of diabetic ketoacidosis, ketonuria, or diabetic hyperosmolar syndrome, osteoporosis, (ii) serum phosphorus (P) > 1.60 mmol/L, (iii) acute infection, (iv) tumors, and (v) pregnant or nursing women, (vi) diagnosis of osteoporosis [According to the WHO criteria (11), osteoporosis was defined as “a BMD that lies 2.5 SD or more below the mean value (a T-score of < -2.5 SD)”. The BMD was checked by using a Lunar iDXA (GE Healthcare, Chicago, IL, USA) at both the whole spine and hip lumbar spine (L1–L4)].

### Anthropometric and Biochemical Analysis

Basic characteristics (sex, age, and family history of T2DM) of the participants were collected. Anthropometric values, such as body weight, height, body mass index (BMI), diastolic blood pressure (DBP), systolic blood pressure (SBP), were measured. Blood samples were drawn between 08:00 a.m. and 09:00 a.m. for laboratory analysis of biochemical variables 25(OH)D, fasting blood glucose (FBG**)**, glycosylated hemoglobin A1c (HbA1c), fasting insulin (FINS), bone alkaline phosphatase (BALP), fasting serum C peptide, insulin-like growth factor-1 (IGF-1), total cholesterol (TC), high-density lipoprotein (HDL-C), triglyceride (TG), low-density lipoprotein (LDL-C), intact parathyroid hormone (iPTH), serum creatinine (SCr), BUN, Ca, P, cystatin C (Cys), homocysteine (Hcy), apolipoprotein-A1 (Apo-A1), and apolipoprotein-B (Apo-B). The anthropometric and biochemical results were analyzed as previously described (8).

### Statistical Analysis

SPSS 19.0 software was used for statistical analysis. Non-normal distribution variables are expressed as median and interquartile ranges. Continuous variables were shown as the mean ± SD. Independent-sample *t*-test was used to compare 25(OH)D, homeostatic model estimates of insulin resistance (HOMA-IR), and glucose metabolism indices (FBG, FINS, and HbA1c), Ca, P, BALP, iPTH, IGF-1 in male and female subjects. Multiple linear regression analysis was used to examine the association between two indices below: (1)serum 25(OH)D concentration and insulin resistance index (HOMA-IR), HOMA-IR analyzed as a dependent variable with the other significantly associated variables [25(OH)D, eGFR, BMI, and age] as independent variables, (2) serum 25(OH)D concentration and bone metabolism indices (Ca, P, calcium-phosphorus production, BALP, and IGF-1), 25 (OH)D, analyzed as the independent variable with the other significantly associated variables (Ca, P, calcium-phosphorus production, BALP, IGF-1, eGFR, BMI, and age) as the dependent variables, (3) HOMA-IR, and bone metabolism indices (Ca, P, calcium-phosphorus production, BALP, and IGF-1), HOMA-IR, analyzed as the independent variable with the other significantly associated variables (Ca, P, calcium-phosphorus production, BALP, IGF-1, eGFR, BMI, and age) as dependent variables. Significance was set at *p* < 0.05.

## Results

### Baseline Characteristics and Bone Metabolism

A total of 109 patients were enrolled in this study. The characteristics of the study subjects and bone metabolism are shown in Table 1. The population had an even sex distribution (55.05% men) and a normal BMI (mean BMI = 24.99 ± 3.30 kg/m^2^) and middle age (mean age = 49.79 ± 13.53 years). The mean 25(OH)D value was 21.10 ± 10.39 ng/ml (52.75 ± 25.98 nmol/L), below the sufficiency cutoff value of 30 ng/ml.

**Table 1:** Characteristics of the study population.

**Characteristics**		***N***
Age (year)^a^	49.79 ± 13.53	109
History (year)^b^	3.00 (0.00–8.00)	109
Male *n* (%)^c^	55.05	60
BMI (kg/m^2^)^a^	24.99 ± 3.30	109
SBp (mmHg)^a^	125.76 ± 14.97	109
DBp (mmol/L)^a^	80.76 ± 9.45	109
FINS (pmol/L)^b^	114.55 (70.78–156.52)	109
FBG (mmol/L)^a^	8.15 ± 3.28	109
HOMA-IR^a^	5.68 ± 2.05	109
Fasting peptide C (μIU/mL)^a^	2.05 ± 1.01	109
25(OH)D (ng/mL)^a^	21.10 ± 10.39	109
Vitamin D deficiency [*n* (%)]^c^	54.13	59
Vitamin D insufficiency [*n* (%)]^c^	24.77	27
Vitamin D sufficiency [*n* (%)]^c^	21.10	23
HbA1c (%)^a^	9.49 ± 2.79	109
TG (mmol/L)^b^	1.65 (1.06–2.63)	109
TC (mmol/L)^a^	5.15 ± 1.13	109
HDL-C (mmol/L)^b^	1.23 (1.10–1.45)	109
LDL-C (mmol/L)^a^	3.08 ± 1.02	109
Apo-A1 (g/L)^b^	1.37 (1.18–1.74)	109
Apo-B (g/L)^a^	1.03 ± 0.32	109
Bun (mmol/L)^b^	4.80 (4.15–5.64)	109
Cr (μmol/L)^b^	56.50 (48.23–69.68)	109
eGFR (mL/min)^b^	111.70 (93.85–130.25)	109
Ca (mmol/L)^a^	2.33 ± 0.13	109
P (mmol/L)^a^	1.36 ± 0.15	109
Calcium [(mg/dL)^2^]^a^	39.24 ± 5.11	109
iPTH (pg/mL)^a^	43.05 ± 14.27	109
BALP (u/L)^a^	184.72 ± 14.77	109
IGF-1 (pg/mL)^a^	276.65 ± 146.52	109
Cys (mg/L)^b^	0.98 (0.91–1.14)	109
Hcy (μmol/L)^a^	9.10 ± 4.11	109

a *Mean ± SD*.

b *Median (interquartile range)*.

c *Percentage*.

*HOMA-IR, homeostatic model estimates of insulin resistance; 25(OH)D, 25-hydroxy vitamin D; TC, total cholesterol; LDL, low-density lipoprotein; Ca, serum calcium; P, serum phosphorus; eGFR, glomerular filtration rate; FINS, fasting blood insulin; TG, triglyceride; HDL, high-density lipoprotein; BUN, blood urea nitrogen; SCr, serum creatinine; HbA1c, glycated hemoglobin A1c; iPTH, intact parathyroid hormone*.

The measurement results of bone metabolism indexes were as follows: Ca 2.33 ± 0.13 mmol/L, P 1.6 ± 0.15 mmol/L, calcium phosphorus production 34.02 ± 9.42 mg/dl, BALP 184.72 ± 14.77 U/L, and IGF-1 276.65 ± 146.52 pg/ml (shown in Table 1).

### Comparison of Men and Women of Insulin Resistance and Glucose (FBG, FINS, and HbA1c) and Bone Metabolic Indices (Ca, P, Calcium-Phosphorus Production, iPTH, BALP, and IGF-1)

The values of serum 25(OH)D, insulin resistance, glucose indices (FBG, FINS levels, and HbA1c), and bone metabolism indices (Ca, P, calcium-phosphorus production, iPTH, BALP, and IGF-1) are shown in Table 2. FINS, FBG, HbA1c, HOMA-IR, 25(OH)D, P, calcium-phosphorus production, BALP, and IGF-1 showed no statistical differences between female and male subjects, but the HbA1c level of female subjects was significantly lower than that of male subjects, while the calcium and iPTH levels of female subjects were significantly higher than those of male subjects.

**Table 2:** Comparison of insulin resistance, glucose (FBG, FINS, and HbA1c) and bone metabolism (Ca, P, calcium, and phosphorus production, iPTH, BALP, and IGF-1) indexes between male and female.

	**Male**	**Female**	***p-*value**
25(OH)D (ng/mL)^a^	20.27 ± 11.43	22.10 ± 8.97	0.364
HOMA-IR^a^	5.79 ± 2.17	5.54 ± 1.90	0.562
HbA1c (%)	10.03 ± 3.02	8.82 ± 2.34	<0.05
FINS (pmol/L)^a^	135.26 ± 76.02	115.90 ± 57.21	0.144
FBG (mmol/L)^a^	7.98 ± 3.76	8.36 ± 2.69	0.545
iPTH (pg/mL)^a^	40.31 ± 14.47	46.40 ± 13.42	<0.05
Ca (mmol/L)^a^	2.29 ± 0.14	2.36 ± 0.12	<0.05
P (mmol/L)^a^	1.35 ± 0.15	1.37 ± 0.13	0.536
Calcium phosphorus production [(mg/dL)^2^]^a^	38.47 ± 5.17	40.18 ± 4.93	0.083
BALP (u/L)^a^	184.84 ± 13.96	184.57 ± 15.85	0.927
IGF-1 (pg/mL)^a^	252.45 ± 138.43	306.28 ± 152.06	0.056

a *Mean ± SD. HOMA-IR, homeostatic model estimates of insulin resistance; 25(OH)D, 25-hydroxy vitamin D; Ca, serum calcium; P, serum phosphorus; FINS, fasting blood insulin; HbA1c, glycated hemoglobin A1c; iPTH, intact parathyroid hormone; BALP, bone alkaline phosphatase; IGF-1, insulin-like growth factor-1*.

### Relationship Between Serum 25(OH)D Concentrations and Bone Metabolism Indices (Ca, P, Calcium-Phosphorus Production, iPTH, BALP, and IGF-1)

**Figure 1:**
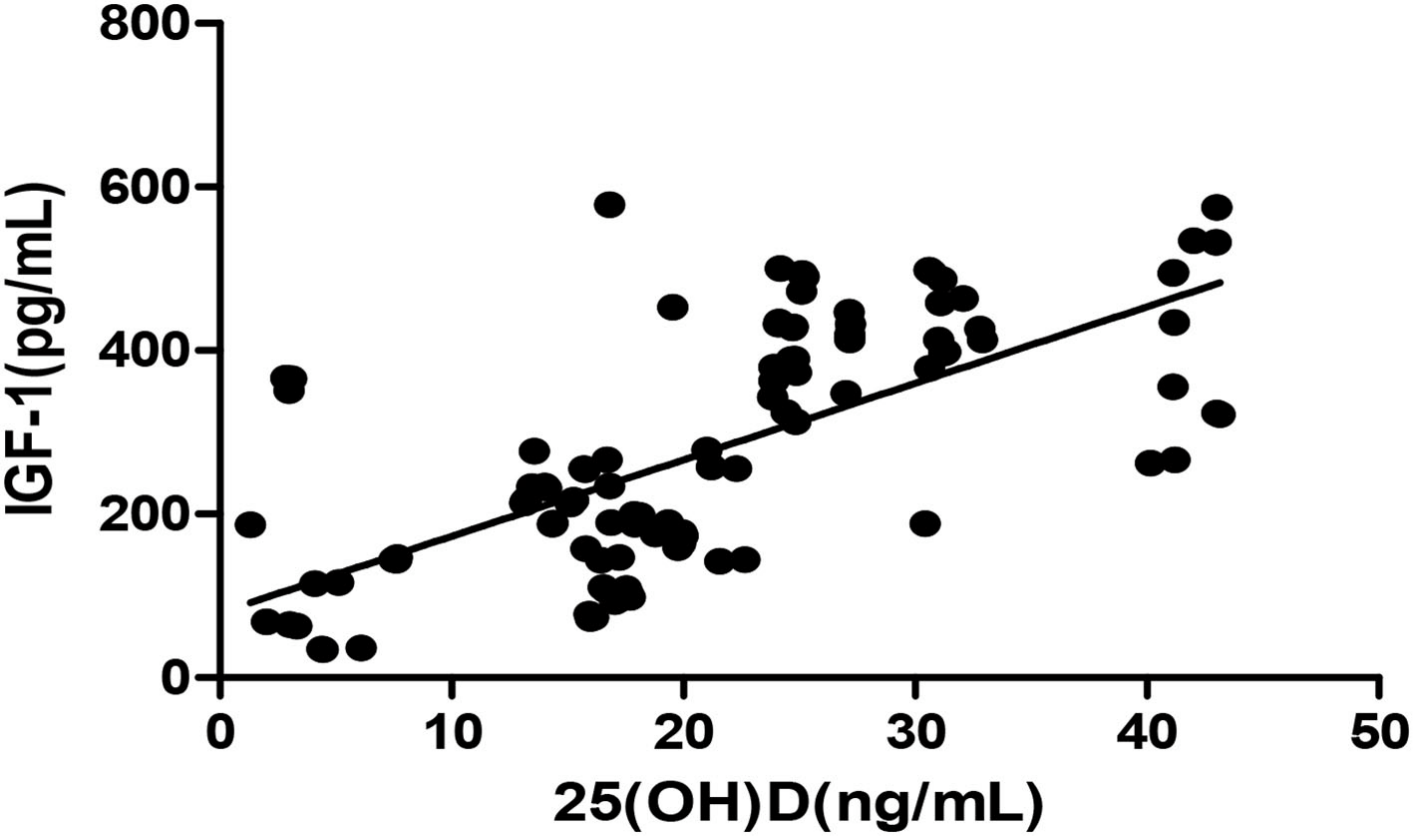
*Pearson* correlation between 25(OH)D and IGF-1 (*r* = 0.664, *p* < 0.05). 25(OH)D, 25-hydroxy vitamin D; TGF-1, insulin-like growth factor-1.

**Figure 2:**
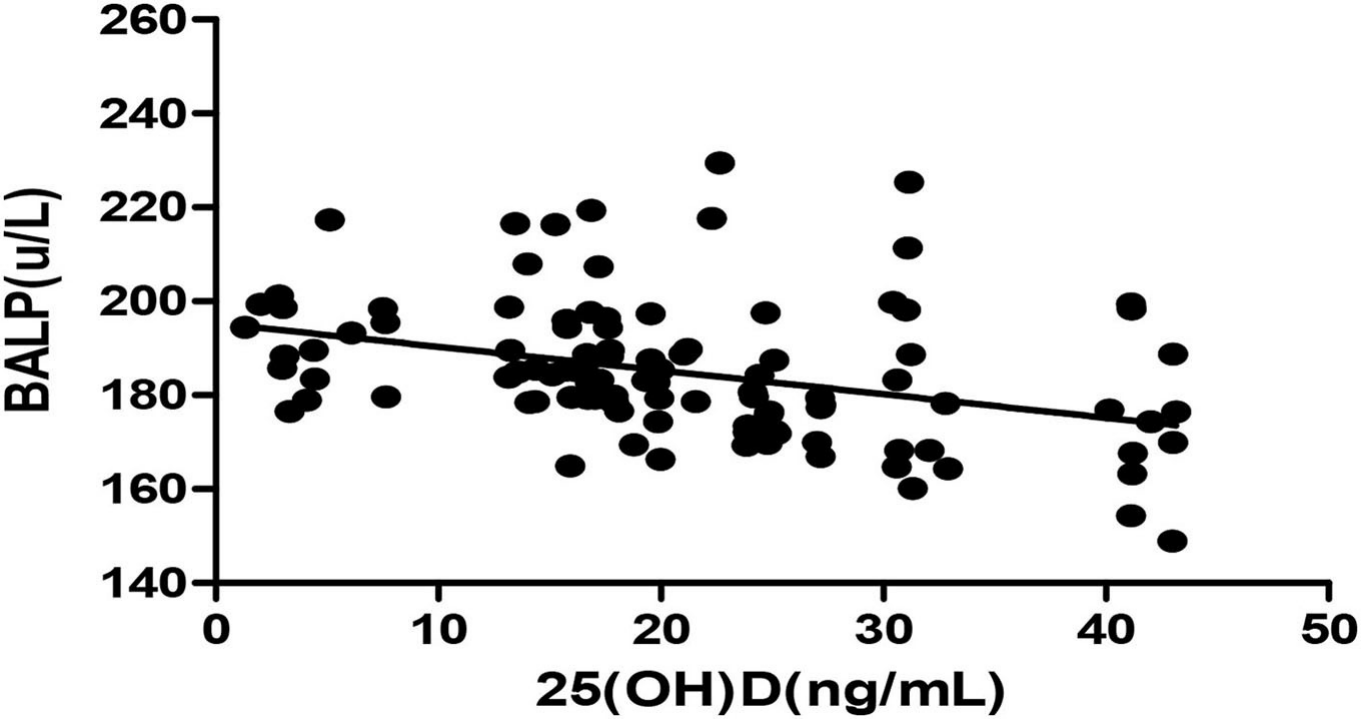
*Pearson* correlation between 25(OH)D and BALP (*r* = −0.355, *p* < 0.05). 25(OH)D, 25-hydroxy vitamin D; BALP, bone alkaline phosphatase.

The *Pearson* correlation coefficient showed a positive correlation between 25(OH)D and IGF-1 (*r* = 0.664, *p* ≤ 0.001; showed in Figure 1), and a negative correlation between 25(OH)D and BALP (*r* = −0. 355, *p* ≤ 0.001; showed in Figure 2). In the multiple linear regression analysis (shown in Table 3), vitamin D status was a predictor of IGF-1 (β = 0.672, *p* ≤ 0.001), BALP (β = −0.369, *p* ≤ 0.001), as the dependent variable, but not Ca, P, calcium-phosphorus production, eGFR, BMI, and age, as independent variables.

**Table 3:** The multiple linear regression analysis of 25(OH)D and bone metabolism indices (Ca, P, calcium-phosphorus production, IGF-1, BALP, and iPTH).

	**β**	***p*-value**
Ca	0.214	0.035
P	0.173	0.079
Calcium phosphorus production	0.233	0.019
IGF-1	0.672	<0.001
BALP	−0.369	<0.001
iPTH	0.108	0.281
Gender	−0.054	0.518

*Variables, such as BMI, age, eGFR, and gender, are adjusted in the linear regression models*.

*BMI, body mass index; eGFR, glomerular filtration rate; Ca, serum calcium; P, serum phosphorus; iPTH, intact parathyroid hormone; BALP, bone alkaline phosphatase; IGF-1, insulin-like growth factor-1*.

### The Multiple Linear Regression Analysis of 25(OH)D and HOMA-IR

**Figure 3:**
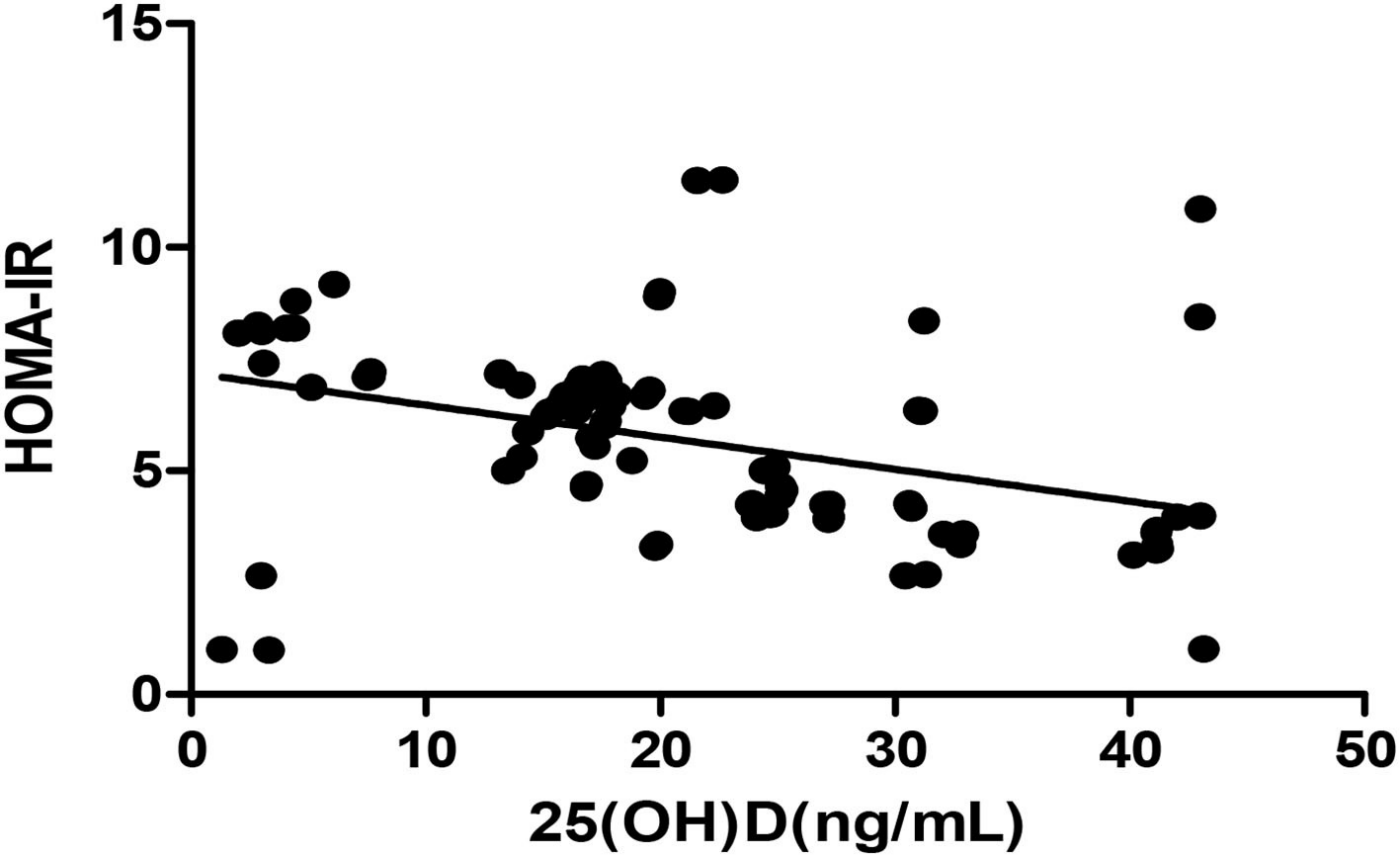
*Pearson* correlation between 25(OH)D and HOMA-IR (*r* = −0.364, *p* < 0.05). HOMA-IR, homeostatic model estimates of insulin resistance; 25(OH)D, 25-hydroxy vitamin D.

The *Pearson* correlation coefficient showed a negative correlation between 25(OH)D concentration and HOMA-IR (*r* = −0.364, *p* ≤ 0.001; shown in Figure 3). In the multiple linear regression analysis (shown in Table 4), vitamin D status was a predictor of HOMA-IR (β = −0.349, *p* ≤ 0.001), as the dependent variable, but not eGFR, BMI, age, and gender, as independent variables.

**Table 4:** The multiple linear regression analysis of 25(OH)D and HOMA-IR.

	**β**	***p-*value**
HOMA-IR	−0.349	<0.001
Age	0.139	0.210
BMI	−0.040	0.688
eGFR	−0.009	0.928
Gender	0.008	0.941

*BMI, body mass index; eGFR, glomerular filtration rate; HOMA-IR, homeostatic model estimates of insulin resistance*.

### The Multiple Linear Regression Analysis of HOMA-IR and Bone Metabolic Indices (Ca, P, Calcium-Phosphorus Production, IGF-1, BALP, and iPTH)

**Figure 4:**
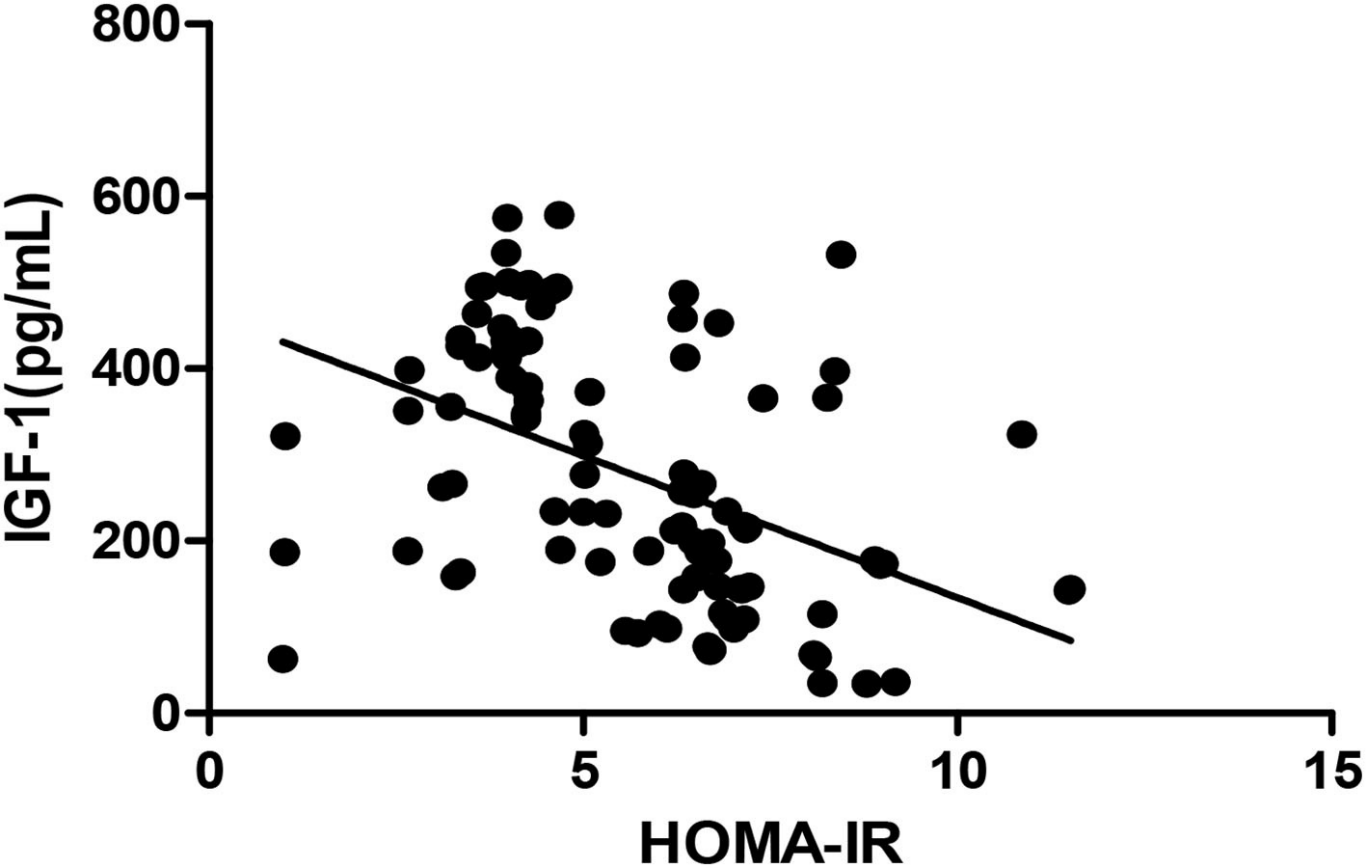
*Pearson* correlation between HOMA-IR and IGF-1 (*r* = −0.460, *p* < 0.05). HOMA-IR, homeostatic model estimates of insulin resistance; TGF-1, insulin-like growth factor-1.

**Figure 5:**
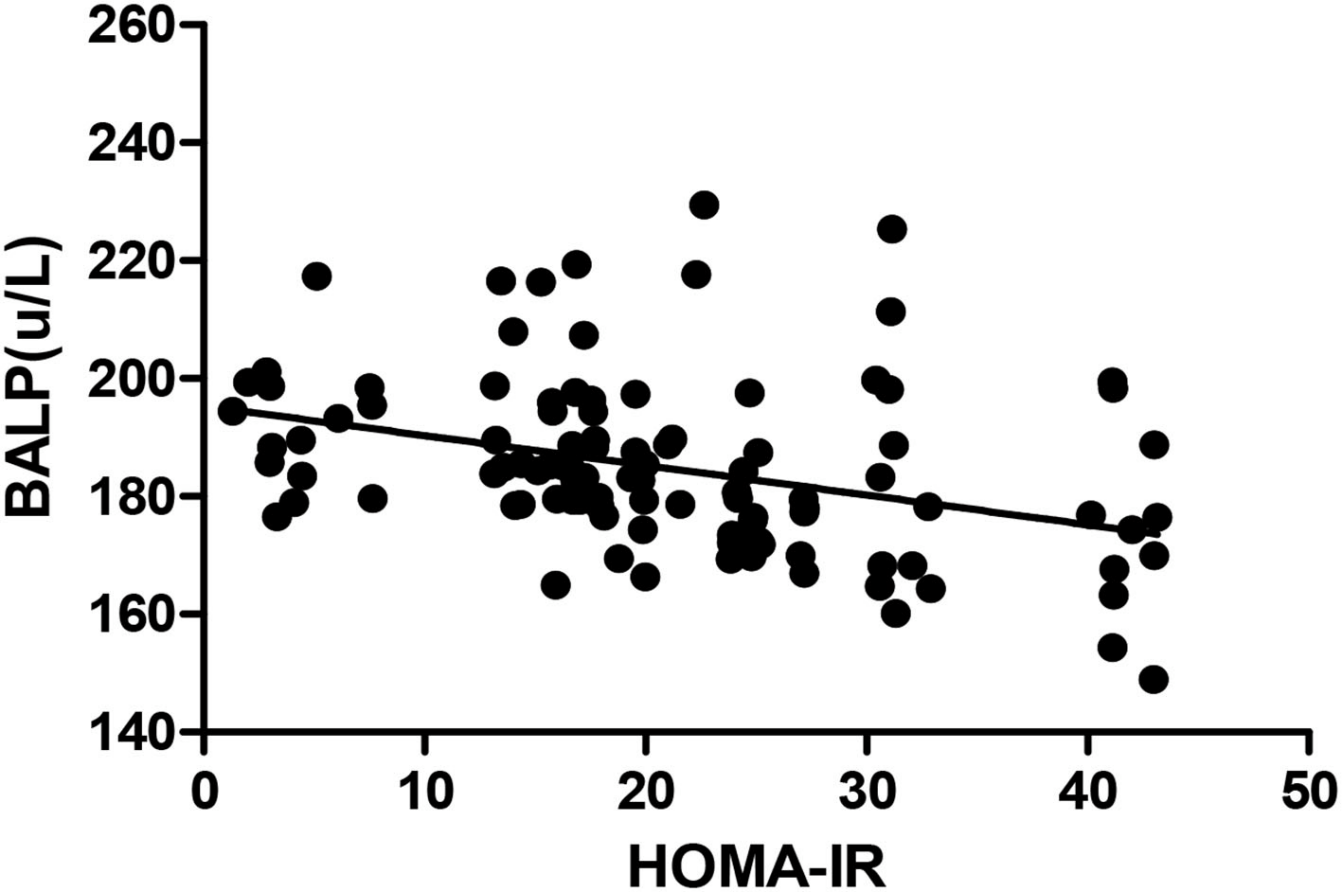
*Pearson* correlation between HOMA-IR and BALP (*r* = 0.342, *p* < 0.05). BALP, bone alkaline phosphatase; HOMA-IR, homeostatic model estimates of insulin resistance.

The *Pearson* correlation coefficient showed that a negative correlation between HOMA-IR and IGF-1 (*r* = −0.460, *p* ≤ 0.001; shown in Figure 4) and a positive correlation with BALP (*r* = 0.342, *p* ≤ 0.001; shown in Figure 5). In the multiple linear regression analysis, HOMA-IR was a predictor of IGF-1 (β = −0.464, *p* ≤ 0.001), BALP (β = 0.344, *p* ≤ 0.001) as the dependent variable, but not Ca, P, calcium-phosphorus production, eGFR, BMI, and age as independent variables (shown in Table 5).

**Table 5:** The multiple linear regression analysis of HOMA-IR and bone metabolic indices (Ca, P, calcium-phosphorus production, IGF-1, BALP, and iPTH).

	**β**	***p-*value**
Ca	−0.004	0.970
P	−0.120	0.225
Calcium phosphorus production	−0.103	0.302
IGF-1	−0.464	<0.001
BALP	0.344	<0.001
iPTH	0.008	0.934
Gender	0.019	0.868

*Variables, such as BMI, age, eGFR, and gender, are adjusted in the linear regression models*.

*BMI, body mass index; eGFR, glomerular filtration rate; Ca, serum calcium; P, serum phosphorus; iPTH, intact parathyroid hormone; BALP, bone alkaline phosphatase; IGF-1, insulin-like growth factor-1*.

## Discussion

In this retrospective cross-section trial, the mean 25(OH)D concentration of all subjects was below the vitamin D sufficient status. We found a significant negative interaction between vitamin D status and HOMA-IR, independent of eGFR, BMI, and age. 25(OH)D concentration showed a negative correlation with BALP and a positive correlation with IGF-1, indicating the decline of osteoblast, risk of osteoporosis. HOMA-IR was significantly negatively associated with IGF-1, positively associated with BALP, adjusted by BMI, eGFR, and age.

The vitamin D receptor being found expressed in many organs in the 1980s, much interest in the extraskeletal effects of vitamin D has attracted considerable scientists (12, 13). Not only regulation of calcium and bone metabolism, but also pleiotropic metabolic roles of vitamin D were found (14). Low vitamin D status was associated with glucose intolerance in man epidemiological studies (15). Abundance research studies suggested that a regulation disruption in vitamin D within the body may result in the development of T2DM (16). In one randomized control trial (RCT), 92 obese adults consumed vitamin D amount to 2,000 IU once a day or 400 mg of calcium twice a day for 16 weeks (17), beta-cell function and insulin secretion were significantly improved in participants supplemented vitamin D, but not in those supplemented calcium alone. However, contrary findings were reported in another RCT (18). Third National Health and Nutrition Examination Survey showed a negative correlation between vitamin D status and morbidity of diabetes, possibly relating with insulin resistance in non-Hispanic whites and Mexican Americans but not non-Hispanic blacks, of which different race might be the reason (19).

It is confirmed that vitamin D and calcium play a vital role in supporting the health of the skeletal system (20). Absorption of calcium in the intestines was reduced because of low levels of vitamin D, leading to an increase of parathyroid hormone levels and bone turnover, subsequently, osteopenia and osteoporosis (21). One research demonstrated that the BMD was less in type 2 diabetic patients who have higher insulin resistance than diabetic patients with less insulin resistance (22).

The growth hormone (GH)/IGF axis is a major determinant of bone mass acquisition. Circulating IGF-1, produced by the liver, is similar to insulin in structure (23) and is responsible for mediating the skeletal growth-promoting actions of GH. IGF-1, acting in a paracrine manner hypothetically, is also produced by bone muscle tissue locally. Along with systemic GH and estradiol, IGF-1 concentration in local bone is regulated by 1,25-dihydroxyvitamin D3, PTH, and other growth factors and cytokines (24). IGF-1, increasing bone matrix deposition and osteoblastic cell recruitment, and decreasing collagen degradation, is considered a key anabolic regulator of bone cell activity (23–25). BALP reflecting bone strength is also assumed as an index for bone quality, quite apart from bone mass (26). The increase of BALP is positively related to osteoporosis. The relationship between the two bone turnover markers, IGF-1 and BALP, with 25(OH)D concentrations indicates that a higher 25(OH)D concentration is good for osteoblast. Contrarily, HOMA-IR may be the risk factor of osteoblast with a significantly negative association with IGF-1, positive one with BALP. In one Korean research, HOMA-IR showed the negative benefits in adolescents' bone mineral content (BMC) (27), which may reasonably assumed the risk of osteoporosis. With the 25(OH)D concentrations decreasing, HOMA-IR indices increased, which showed the deteriorating insulin sensitivity. The same result was shown in SIR—Studies in animals that vitamin D deficiency is correlated to impaired insulin sensitivity, and that vitamin D supplementation increased insulin secretion (28). Although all the subjects were non-osteoporosis patients with T2DM in our research, more than half of the subjects (54.13%) were with vitamin D deficiency. It is commonly agreed that vitamin D is responsible to maintain normal levels of Ca and P, vitamin D deficient subjects indicated a high risk of osteoporosis.

As a result, 25(OH)D concentrations are negatively related to both insulin resistance and bone turnover, which increases the risk of osteoporosis. As reported that supplementation of vitamin D ameliorated insulin resistance in patients with T2DM, it is thought that vitamin D deficiency results in a decrease of insulin sensitivity (29). Some vitro trials also proved that vitamin D regulated the insulin release directly or via vitamin D receptor (30–33). As in our research, regardless of age, BMI, eGFR, HOMA-IR negative related to IGF-1 and positively related to BALP, we assumed that it is possible insulin resistance decreases the bone turnover, raises the risk of osteoporosis. The casual relationship should be conferred by further research. We speculate that insulin resistance could be one of the mechanisms of osteoporosis in T2DM.

Calcium-phosphorus production, phosphorus, BALP, IGF-1, HOMA-IR, FINS, and FBG show no difference between men and women, except levels of calcium and iPTH in men lower than that in women, probably effect in climacteric female subjects.

As to our knowledge, this is the first research to discuss the association between vitamin D status, insulin resistance, and bone metabolism in Chinese non-osteoporosis patients with T2DM.

## Limitation

As the limit capacity of the fund, only two indices of bone turnover were used to evaluate the bone metabolism status. However, Bandeira et al. suggested since in most cases bone formation mirrors bone resorption, and vice versa, a single bone turnover marker could be used in clinical practice, considering the cost, sometimes (34). Considering the seasonal effects on vitamin D status, the recruitment of the subjects was restricted to 3 months. The number of recruited subjects was not large enough to analyze in subgroup to adjust the effect of female climacteric. As the nature of the observation trial, the casual relationship could not be proved.

## Conclusion

As the 25(OH)D concentrations were decreased, HOMA-IR and BALP were increased, and IGF-1 was decreased. 25(OH)D concentrations are negatively related to both insulin resistance and bone turnover, which increase the risk of osteoporosis. Whether vitamin D supplement is needed in pre-osteoporosis stage patients with T2DM is required more research to clarify.

## Data Availability Statement

The original contributions presented in the study are included in the article/supplementary material, further inquiries can be directed to the corresponding author/s.

## Ethics Statement

The studies involving human participants were reviewed and approved by the Ethics Committee of Xiamen Second Hospital Affiliated Xiamen Medical College. Written informed consent for participation was not required for this study in accordance with the national legislation and the institutional requirements.

## Author Contributions

JZ established the original concept and wrote the first draft. YL, DLa, and DLu collected clinical blood samples. ZL, JK, YX, and SC conducted experiments. All authors discussed the results and edited the manuscript.

## Funding

This work was supported by the project of 2019 Fujian Province Health Research Talent Cultivation Project Medical Innovation (Grant No. 2019-CXB-38) and the project of 2018 Outstanding Young Scientific Research Talents in Colleges and Universities of Fujian Province (Grant No. Min Education and Scientific Department [2018] 47).

## Conflict of Interest

The authors declare that the research was conducted in the absence of any commercial or financial relationships that could be construed as a potential conflict of interest.

## Publisher's Note

All claims expressed in this article are solely those of the authors and do not necessarily represent those of their affiliated organizations, or those of the publisher, the editors and the reviewers. Any product that may be evaluated in this article, or claim that may be made by its manufacturer, is not guaranteed or endorsed by the publisher.

## References

[1] Hossain P Kawar B El Nahas M. Obesity and diabetes in the developing world–a growing challenge. N Engl J Med. (2007) 356:213–5. 10.1056/NEJMp068177 17229948

[2] Cooper C Cole ZA Holroyd CR Earl SC Harvey NC Dennison EM. Secular trends in the incidence of hip and other osteoporotic fractures. Osteoporos Int. (2011) 22:1277–88. 10.1007/s00198-011-1601-6 21461721 PMC3546313

[3] Oden A McCloskey EV Kanis JA Harvey NC Johansson H. Burden of high fracture probability worldwide: secular increases 2010-2040. Osteoporos Int. (2015) 26:2243–8. 10.1007/s00198-015-3154-6 26018089

[4] Kumeda Y Inaba M Nishizawa Y. Secondary osteoporosis and its treatment–diabetes mellitus. Nihon Rinsho. (1998) 56:1579–86. 9648485

[5] Bachali S Dasu K Ramalingam K Naidu JN. Vitamin d deficiency and insulin resistance in normal and type 2 diabetes subjects. Indian J Clin Biochem. (2013) 28:74–8. 10.1007/s12291-012-0239-2 24381426 PMC3547453

[6] Kostoglou-Athanassiou I Athanassiou P Gkountouvas A Kaldrymides P. Vitamin D and glycemic control in diabetes mellitus type 2. Ther Adv Endocrinol Metab. (2013) 4:122–8. 10.1177/2042018813501189 23997931 PMC3755528

[7] Liang W Luo Z Ge S Li M Du J Yang M. Oral administration of quercetin inhibits bone loss in rat model of diabetic osteopenia. Eur J Pharmacol. (2011) 670:317–24. 10.1016/j.ejphar.2011.08.014 21914440

[8] Zhang J Ye J Guo G Lan Z Li X Pan Z. Vitamin D status is negatively correlated with insulin resistance in chinese type 2 diabetes. Int J Endocrinol. (2016) 2016:1794894. 10.1155/2016/1794894 27413370 PMC4931076

[9] Levey AS Bosch JP Lewis JB Greene T Rogers N Roth D. A more accurate method to estimate glomerular filtration rate from serum creatinine: a new prediction equation. Modification of Diet in Renal Disease Study Group. Ann Intern Med. (1999) 130:461–70. 10.7326/0003-4819-130-6-199903160-00002 10075613

[10] Alberti KG Zimmet PZ. Definition, diagnosis and classification of diabetes mellitus and its complications. Part 1: diagnosis and classification of diabetes mellitus provisional report of a WHO consultation. Diabet Med. (1998) 15:539–53. 10.1002/(SICI)1096-9136(199807)15:7<539::AID-DIA668>3.0.CO;2-S 9686693

[11] Grynpas M. Age and disease-related changes in the mineral of bone. Calcif Tissue Int. (1993) 53:S57–64. 10.1007/BF01673403 8275381

[12] Holick MF. The vitamin D deficiency pandemic and consequences for nonskeletal health: mechanisms of action. Mol Aspects Med. (2008) 29:361–8. 10.1016/j.mam.2008.08.008 18801384 PMC2629072

[13] Holick MF. Vitamin D: extraskeletal health. Endocrinol Metab Clin North Am. (2010) 39:381–400. 10.1016/j.ecl.2010.02.016 20511059

[14] Nagpal S Na S Rathnachalam R. Noncalcemic actions of vitamin D receptor ligands. Endocr Rev. (2005) 26:662–87. 10.1210/er.2004-0002 15798098

[15] Pittas AG Lau J Hu FB Dawson-Hughes B. The role of vitamin D and calcium in type 2 diabetes. A systematic review and meta-analysis. J Clin Endocrinol Metab. (2007) 92:2017–29. 10.1210/jc.2007-0298 17389701 PMC2085234

[16] Florez H Troen BR. Do vitamin D levels influence the risk of diabetes mellitus and play a role in healthier aging? J Am Geriatr Soc. (2011) 59:1957–9. 10.1111/j.1532-5415.2011.03592.x 22091507

[17] Mitri J Dawson-Hughes B Hu FB Pittas AG. Effects of vitamin D and calcium supplementation on pancreatic beta cell function, insulin sensitivity, and glycemia in adults at high risk of diabetes: the Calcium and Vitamin D for Diabetes Mellitus (CaDDM) randomized controlled trial. Am J Clin Nutr. (2011) 94:486–94. 10.3945/ajcn.111.011684 21715514 PMC3142723

[18] Davidson MB Duran P Lee ML Friedman TC. High-dose vitamin D supplementation in people with prediabetes and hypovitaminosis D. Diabetes Care. (2013) 36:260–6. 10.2337/dc12-1204 23033239 PMC3554269

[19] Scragg R Sowers M Bell C Third National H, Nutrition Examination S. Serum 25-hydroxyvitamin D, diabetes, and ethnicity in the Third National Health and Nutrition Examination Survey. Diabetes Care. (2004) 27:2813–8. 10.2337/diacare.27.12.2813 15562190

[20] Ross AC Manson JE Abrams SA Aloia JF Brannon PM Clinton SK. The 2011 dietary reference intakes for calcium and vitamin D: what dietetics practitioners need to know. J Am Diet Assoc. (2011) 111:524–7. 10.1016/j.jada.2011.01.004 21443983

[21] van den Bergh JP Bours SP van Geel TA Geusens PP. Optimal use of vitamin D when treating osteoporosis. Curr Osteoporos Rep. (2011) 9:36–42. 10.1007/s11914-010-0041-0 21113692 PMC3026680

[22] Arikan S Tuzcu A Bahceci M Ozmen S Gokalp D. Insulin resistance in type 2 diabetes mellitus may be related to bone mineral density. J Clin Densitom. (2012) 15:186–90. 10.1016/j.jocd.2011.11.005 22321655

[23] Van Wyk JJ Smith EP. Insulin-like growth factors and skeletal growth: possibilities for therapeutic interventions. J Clin Endocrinol Metab. (1999) 84:4349–54. 10.1210/jcem.84.12.6201 10599687

[24] Yakar S Rosen CJ Beamer WG Ackert-Bicknell CL Wu Y Liu JL. Circulating levels of IGF-1 directly regulate bone growth and density. J Clin Invest. (2002) 110:771–81. 10.1172/JCI0215463 12235108 PMC151128

[25] Mohan S Baylink DJ. IGF-binding proteins are multifunctional and act via IGF-dependent and -independent mechanisms. J Endocrinol. (2002) 175:19–31. 10.1677/joe.0.1750019 12379487

[26] Ohta H. Bone and bone related biochemical examinations. Bone and collagen related metabolites Bone-specific alkaline phosphatase. Clin Calcium. (2006) 16:1022–8. 16751700

[27] Park SW Nam GE Jung DW Yoon SJ Han K Park YG. Association of lipid parameters and insulin resistance with bone health in South Korean adolescents. Osteoporos Int. (2016) 27:635–42. 10.1007/s00198-015-3306-8 26329100

[28] Tai K Need AG Horowitz M Chapman IM. Vitamin D, glucose, insulin, and insulin sensitivity. Nutrition. (2008) 24:279–85. 10.1016/j.nut.2007.11.006 18187309

[29] Kayaniyil S Vieth R Retnakaran R Knight JA Qi Y Gerstein HC. Association of vitamin D with insulin resistance and beta-cell dysfunction in subjects at risk for type 2 diabetes. Diabetes Care. (2010) 33:1379–81. 10.2337/dc09-2321 20215450 PMC2875459

[30] Bischoff HA Borchers M Gudat F Duermueller U Theiler R Stahelin HB. *In situ* detection of 1,25-dihydroxyvitamin D3 receptor in human skeletal muscle tissue. Histochem J. (2001) 33:19–24. 10.1023/A:1017535728844 11352397

[31] Norman AW. Minireview: vitamin D receptor: new assignments for an already busy receptor. Endocrinology. (2006) 147:5542–8. 10.1210/en.2006-0946 16946007

[32] Li J Byrne ME Chang E Jiang Y Donkin SS Buhman KK. 1alpha,25-Dihydroxyvitamin D hydroxylase in adipocytes. J Steroid Biochem Mol Biol. (2008) 112:122–6. 10.1016/j.jsbmb.2008.09.006 18840526 PMC2602794

[33] Alvarez JA Ashraf A. sRole of vitamin d in insulin secretion and insulin sensitivity for glucose homeostasis. Int J Endocrinol. (2010) 2010:351385. 10.1155/2010/351385 20011094 PMC2778451

[34] Bandeira F Costa AG Soares Filho MA Pimentel L Lima L Bilezikian JP. Bone markers and osteoporosis therapy. Arq Bras Endocrinol Metabol. (2014) 58:504–13. 10.1590/0004-2730000003384 25166041

